# Investigating the benefits of molecular profiling of advanced non-small cell lung cancer tumors to guide treatments

**DOI:** 10.18632/oncotarget.24375

**Published:** 2018-02-01

**Authors:** Costi Alifrangis, Philip Carter, Biancastella Cereser, Pramodh Chandrasinghe, Lisa Del Bel Belluz, Eric Lim, Nina Moderau, Fotini Poyia, Neha Tabassum, Hua Zhang, Jonathan Krell, Justin Stebbing

**Affiliations:** ^1^ Department of Oncology, University College Hospital, London, UK; ^2^ Department of Surgery and Cancer, Imperial College, London, UK; ^3^ Department of Surgery, University of Kelaniya, Kelaniya, Sri Lanka; ^4^ National Heart and Lung Institute, Imperial College, London, UK; ^5^ Department of Medical Oncology, Dana-Farber Cancer Institute, Boston, MA, USA

**Keywords:** tumor profiling, lung cancer, NSCLC, non-small cell lung cancer, cancer treatment

## Abstract

In this study we utilized data on patient responses to guided treatments, and we evaluated their benefit for a non-small cell lung cancer cohort. The recommended therapies used were predicted using tumor molecular profiles that involved a range of biomarkers but primarily used immunohistochemistry markers. A dataset describing 91 lung non-small cell lung cancer patients was retrospectively split into two. The first group's drugs were consistent with a treatment plan whereby all drugs received agreed with their tumor's molecular profile. The second group each received one or more drug that was expected to lack benefit.

We found that there was no significant difference in overall survival or mortality between the two groups. Patients whose treatments were predicted to be of benefit survived for an average of 402 days, compared to 382 days for those that did not (*P* = 0.7934). In the matched treatment group, 48% of patients were deceased by the time monitoring had finished compared to 53% in the unmatched group (*P* = 0.6094). The immunohistochemistry biomarker for the ERCC1 receptor was found to be a marker that could be used to predict future survival; ERCC1 loss was found to be predictive of poor survival.

## INTRODUCTION

Lung cancer is the most common cancer in the world. Non-small cell lung cancer (NSCLC) is the fourth most prevalent cancer in Europe causing 13% of all cases, and the third most frequent in the UK. Lung cancer incidence is strongly linked with age, in the UK about 61% of cases are diagnosed in people aged 70 and over. Smoking is by far the leading risk factor; about 80% of deaths are thought to result from smoking. In the UK, the long-term likelihood of developing lung cancer for men is about 1 in 13, and for women it is around 1 in 17 [1].

Metastatic NSCLC has traditionally had poor outcomes. Metastatic or stage IV NSCLC, has a 5-year survival rate of about 1%. In the metastatic disease, treatment with cytotoxic chemotherapy is only for palliative purposes, and prior to new advances the median survival benefit of a course of platinum-based chemotherapy was approximately two months [2].

In recent years, targeted treatments and immunotherapy (alone or in combination) have been used to treat lung cancer. Following identification of activating mutations in the *EGFR* gene in a subset of patients with NSCLC, tremendous advances have been made in the use of predictive biomarkers to stratify patients according to the oncogenes thought to be driving their malignancy. This is most commonly applicable in non-smokers, and currently stratification presents a treatment modality in approximately 10–20% of patients, depending on ethnicity. Somatically acquired mutations in the *EGFR* gene, or the acquisition of a fusion transcript of the *EML4-ALK* or *ROS1* oncogenes, have been associated with heightened responses to allosteric tyrosine kinase inhibitors that are specific to the resultant aberrant kinase. Second and third generation kinase inhibitors are now in routine clinical use in these subsets of NSCLC. However, if a patient lacks a defined mutation in one of these proteins, it is unclear whether they will gain from additional molecular testing and/or therapy selection.

Previous studies have demonstrated the benefit of tumor profiling [3–5] in other types of tumor. Here we used data from Caris Life Sciences to see if tumor molecular profiling including identification of genetic mutations led to better clinical outcomes when used to give treatment recommendations in a NSCLC cohort. We also looked at the impact of profiling on drug usage.

## RESULTS

Historical information about treatments and clinical outcomes for 91 advanced stage NSCLC patients taken from the Caris CODE database was divided into two groups. This was according to if their treatments had matched recommendations predicted from their tumor's molecular profile; the recommendations are stated to be mostly obtained from previous research identified in the literature.

Treatments were largely related to chemotherapy; no patients in this cohort received active immunotherapy; and no patients received checkpoint inhibitors prior to profiling. Chemotherapy predominated, with only three patients receiving no chemotherapy. The 88 patients that received it were given 237 chemotherapies (not including repeats of the same treatment in a patient). Targeted therapies (not including moAbs) were received by 20 patients, who received 21 of these treatments, while moAbs were given to 32 patients (34 therapies: 31 bevacizumab and 3 cetuximab).

**Figure 1:**
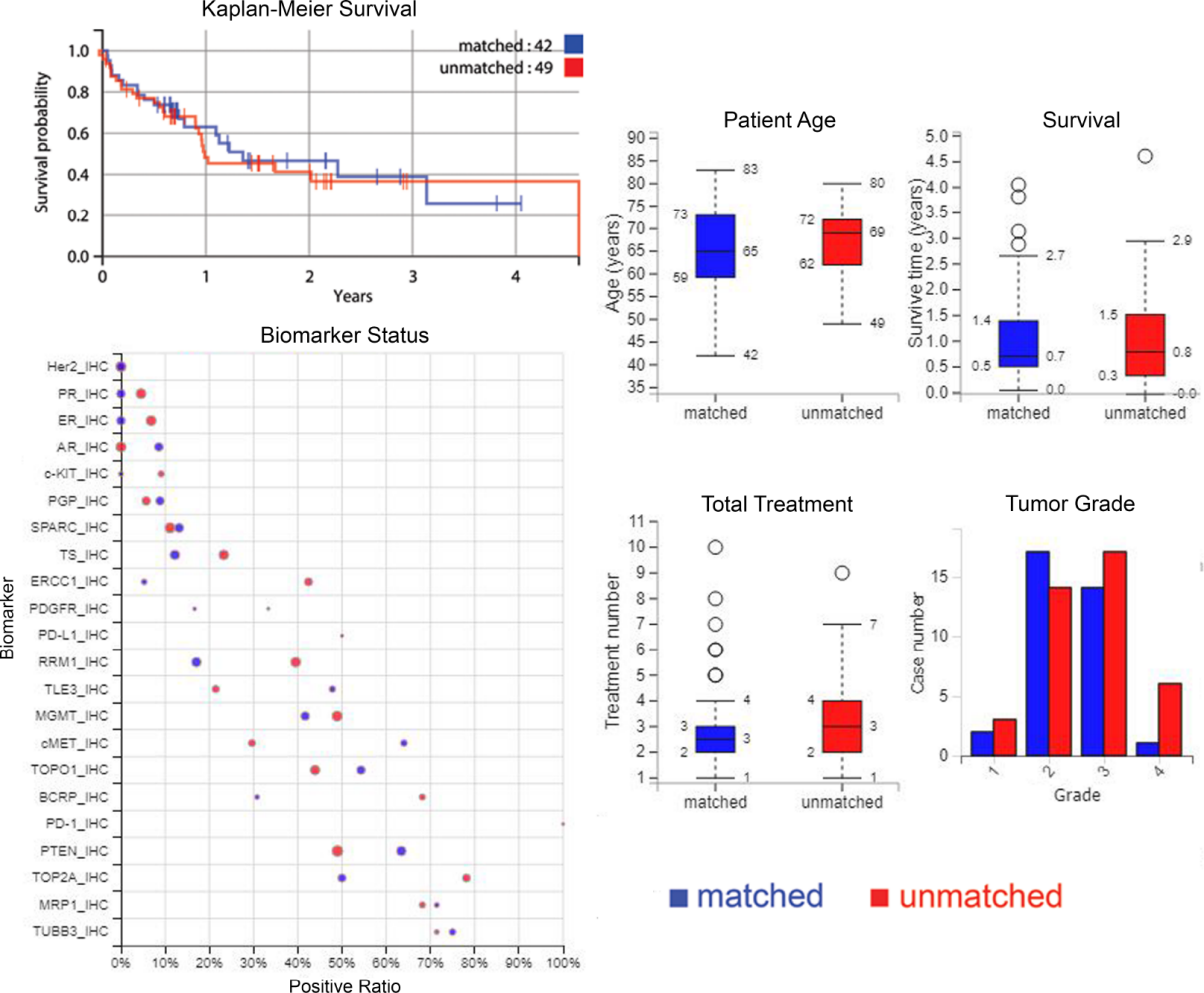
Plots of survival, biomarkers, and patient, treatment and tumour characteristics *Upper-left*: a Kaplan–Meier curve showing overall survival from time of profiling for matched versus unmatched treatment groups. *Lower-left*: comparison of biomarkers between matched and unmatched where positive ratio gives the percentage of cases that have “positive” biomarker results i.e. for IHC tests, positive is protein expression above a certain threshold, and for sequencing biomarkers, positive is a gene mutation. The size of the circle represents the number of cases. *On the right*: age of patients, survival time, treatment numbers, and grade of samples, where blue is matched patients and red is unmatched patients.

In the matched treatment group, 42 patients were given one or more drugs after sample collection for profiling that agreed with their tumor's molecular profile and no treatments predicted to lack benefit. In the unmatched treatment group, 49 patients received one or more drugs that were expected to lack benefit after the time of collection for profiling. Information about the demographics of both groups are summarized in Tables 1 and 2, and Figure 1 (see the plots on the right), along with information about the tumors.

**Table 1:** Patient ages for the two treatment groups

Age	Matched	Unmatched
20–29	0	0
30–39	0	0
40–49	4	1
50–59	7	8
60–69	17	19
70–79	9	19
80–89	5	2

**Table 2:** Summary of patients’ information comparing the matched and unmatched subsets against both combined

Group	Patient & Tumor Information						
	Age	Ethnicity	Histology	Grade	Stage	Survival (Days)	Mortality (%)
*All patients (91)*	66.1	White: 74; Black/African American: 8; Asian: 6; Hawaiian/Pacific Islander: 2; Other/Unknown: 1	Adenocarcinoma, NOS: 50; Squamous cell carcinoma, NOS: 19; Non-small cell carcinoma: 9; Adenosquamous carcinoma: 3; Papillary adenocarcinoma, NOS: 2; Squamous cell carcinoma, keratinizing, NOS: 2; Large cell carcinoma, NOS: 2; Signet ring cell carcinoma: 1; Carcinoma, undifferentiated, NOS: 1; Mucinous adenocarcinoma: 1; Adenocarcinoma with mixed subtypes: 1	Grade 3/ Poorly differentiated: 31 (34%); Grade 2/Moderately differentiated: 31 (34%); Unknown/Not determined: 13 (14%); Grade 4/ Undifferentiated: 7 (8%); Grade 1/Well differentiated: 5 (6%); None/Not applicable: 4 (4%)	IV: 43 (47%); III no IIIC: 25 (28%); II: 14 (15%); I: 7 (8%); Unknown: 2 (2%)	391	51
*Matched only (42)*	65.1	White: 34; Asian: 4; Black/African American: 2; Hawaiian/Pacific Islander: 1; Other/Unknown: 1	Adenocarcinoma, NOS: 25; Squamous cell carcinoma, NOS: 7; Non-small cell carcinoma: 5; Adenosquamous carcinoma: 1; Signet ring cell carcinoma: 1; Papillary adenocarcinoma, NOS: 1; Adenocarcinoma with mixed subtypes: 1; Mucinous adenocarcinoma: 1	Grade 2/Moderately differentiated: 17 (41%); Grade 3/ Poorly differentiated: 14 (33%); Unknown/Not determined: 6 (14%); None/Not applicable: 2 (5%); Grade 1/Well differentiated: 2 (5%); Grade 4/ Undifferentiated: 1 (2%)	IV: 17 (40%); III no IIIC: 13 (31%); II: 7 (17%); I: 4 (10%); Unknown: 1 (2%)	402	48
*Unmatched (49)*	67.1	White: 40; Black/African American: 6; Asian: 2; Hawaiian/Pacific Islander: 1	Adenocarcinoma, NOS: 25; Squamous cell carcinoma, NOS: 12; Non-small cell carcinoma: 4; Squamous cell carcinoma, keratinizing, NOS: 2; Adenosquamous carcinoma: 2; Large cell carcinoma, NOS: 2; Carcinoma, undifferentiated, NOS: 1; Papillary adenocarcinoma, NOS: 1	Grade 3/ Poorly differentiated: 17 (35%); Grade 2/Moderately differentiated: 14 (29%); Unknown/Not determined: 7 (14%); Grade 4/ Undifferentiated: 6 (12%); Grade 1/Well differentiated: 3 (6%); None/Not Applicable: 2 (4%)	IV: 26 (53%); III no IIIC: 12 (25%); II: 7 (14%); I: 3 (6%); Unknown: 1 (2%)	382	53

**Figure 2:**
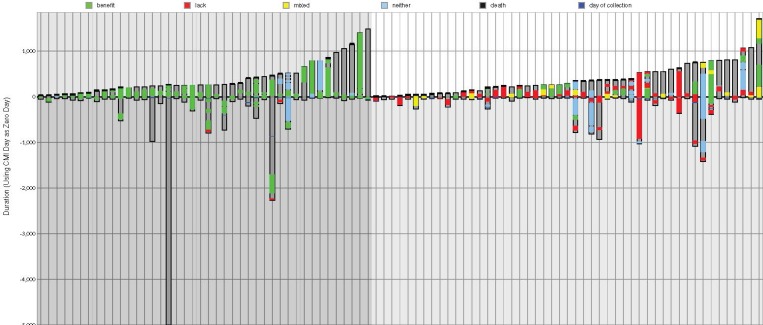
Patients' treatment schedules and outcomes Treatment plans for the 42 matched patients shown in ascending post-profiling survival time are shown on the left (darker background), and for the 49 unmatched patients are on the right (lighter background). The ordinate is time in days where the axis is the time of profiling. Gray = total time monitored from diagnosis; a black line at the top of a column is death; green = time on a drug of benefit; red = lack of benefit treatment; yellow = combination therapy including benefit and lack of benefit drugs; blue = neutral therapy (neither benefit nor lack of benefit).

The average survival of the two groups is compared in Figure 2, in which each bar displayed in the graph denotes an individual lung cancer patient and their treatments; 42 matched (on the left) and 49 unmatched patients (on the right) are shown overall.

Table 3 lists the drugs most frequently given for: all patients (total drugs and total treatment periods are shown separately); the matched and unmatched groups; the most often given drugs predicted to be of benefit; lacking benefit; and neither of these. The number of patients treated with a drug is shown in the first column, and the number of continuous treatment periods is shown in all other columns, i.e. treatments of the same patient with intervening periods are counted separately. The drugs given to the most number of patients were carboplatin (74 patients), pemetrexed disodium (41), docetaxel (35), and bevacizumab (31). Patients received an average of 3.73 drugs, of which 34% (115 treatment periods) were expected to be of benefit, 24% (80) lacked benefit, and 42% (144) being neither of these types. Matched patients on average had 3.5 drugs-52.7% (1.86 treatments) of these were of type benefit, 2% (0.07) lacked benefit, and 45.3% (1.60) being neither of these. Unmatched patients had 3.9 drug treatments on average; 19.4% (0.76 drugs) of these were classed as of benefit, 40.3% (1.57) lacked benefit, and 40.3% (1.57) neither. Of patients in the unmatched set 51% had at least one beneficial drug, and 20% received two or more drugs of benefit. When comparing the drugs given after profiling in the matched group to those given when including the time before profiling also, docetaxel was given less often after profiling while erlotinib was given more frequently.

**Table 3:** The most frequently given drugs by treatment group and those predicted to be of benefit, lacking benefit, or neither, listed in descending order

Number of Patients Treated	Most Frequently Administered Drugs (Total Treatment Periods)							
All patients treated	All patients-treatment periods	Matched only patients, all treatments	Matched, after profiling treatments only	Unmatched patients, all treatments	Unmatched, after profiling treatments only	Drugs predicted of benefit	Drugs predicted to lack benefit	Drugs with no prediction (neither of benefit or lack of benefit)
carboplatin - 74 patients	carboplatin: 85	carboplatin: 36	pemetrexed disodium: 13	carboplatin: 49	pemetrexed disodium: 13	pemetrexed disodium: 23	carboplatin: 31	bevacizumab: 35
pemetrexed disodium - 41 patients	pemetrexed disodium: 48	pemetrexed disodium: 21	erlotinib hydrochloride: 11	pemetrexed disodium: 27	bevacizumab: 12	carboplatin 22	pemetrexed disodium: 10	carboplatin: 30
docetaxel - 35 patients	docetaxel: 39	docetaxel: 20	carboplatin: 10	bevacizumab: 22	carboplatin: 11	erlotinib hydrochloride: 17	docetaxel; gemcitabine hydrochloride; cisplatin: 8	pemetrexed disodium; docetaxel: 15
bevacizumab - 31 patients	bevacizumab: 38	bevacizumab: 16	bevacizumab: 8	docetaxel: 19	gemcitabine hydrochloride: 10	docetaxel: 16	-	-
paclitaxel - 23 patients	paclitaxel; erlotinib hydrochloride: 24	erlotinib hydrochloride: 13	docetaxel: 5	paclitaxel: 17	erlotinib hydrochloride; nab-paclitaxel: 6	gemcitabine hydrochloride: 9	-	paclitaxel: 13
gemcitabine hydrochloride - 21 patients	-	paclitaxel; cisplatin :7	capecitabine; cisplatin; gemcitabine hydrochloride: 2	gemcitabine hydrochloride: 16	-	paclitaxel; nab-paclitaxel: 7	erlotinib hydrochloride: 6	cisplatin; vinorelbine tartrate: 6
cisplatin; erlotinib hydrochloride - 17 patients	gemcitabine hydrochloride: 22	-	-	cisplatin: 13	cisplatin; docetaxel: 4	-	paclitaxel: 4	-
-	cisplatin: 20	gemcitabine hydrochloride: 6	-	erlotinib hydrochloride: 11	-	cisplatin: 5	cetuximab; nab-paclitaxel: 2	etoposide: 5
nab-paclitaxel - 10 patients	nab-paclitaxel: 11	nab-paclitaxel: 4	vinorelbine tartrate; fluorouracil; cetuximab; irinotecan hydrochloride; afatinib dimaleate; paclitaxel; nab-paclitaxel; leucovorin calcium: 1	nab-paclitaxel: 7	vinorelbine tartrate: 3	bevacizumab; crizotinib: 2	-	gemcitabine hydrochloride: 4
vinorelbine tartrate - 6 patients	vinorelbine tartrate: 6	fluorouracil; cetuximab; capecitabine; etoposide: 2	-	vinorelbine tartrate: 5	crizotinib; paclitaxel 2	-	crizotinib: 1	fluorouracil: 2

The most commonly given drugs that when given were expected to be of benefit were pemetrexed disodium (23 treatment periods), carboplatin (22), erlotinib hydrochloride (17), and docetaxel (16). The most frequently given lack of benefit drug was carboplatin (31). The neither category makes up 45% of drugs administered in the matched group, compared to 40% in the unmatched group. The most popular agents in the neither category were bevacizumab (35 treatments, i.e. 10% of drugs overall) and carboplatin (30 times). Interestingly, drugs most commonly used in metastatic NSCLC, e.g. platinums, were given at similar rates independently of whether they were predicted to help.

Overall the drug given most often was carboplatin (for 85 time periods over 7845 days in total), although following profiling, pemetrexed disodium was given the most number of times (26 time periods over 2688 days in total).

Patients in the matched group on average survived for 402 days after monitoring, compared to 382 days for patients in the unmatched group (*P* = 0.7934). In the matched group 48% of patients were deceased when monitoring ended, compared to 53% of the unmatched group of patients (*P* = 0.6094). These differences are therefore minor and not statistically significant. A Kaplan-Meier curve showing the overall survival for the matched group compared to the unmatched is also shown in Figure 1 (upper-left).

Patients who were given more than one drug that lacked benefit were found to have a worse overall survival (OS) than patients who received only a single drug of this type (466.3 versus 318.7 days respectively).

**Figure 3:**
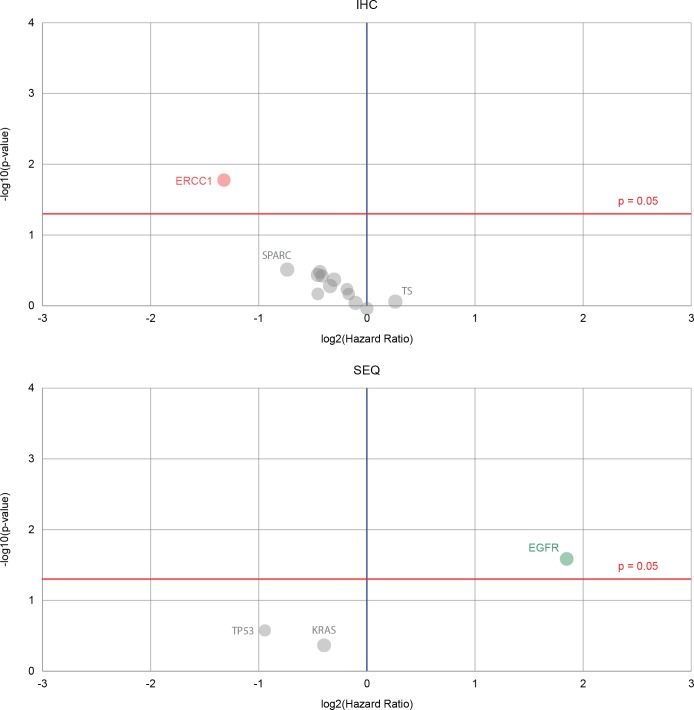
Volcano plots that show biomarkers’ prognostic value for a lung cancer dataset Red dots indicate the hazard rate of a positive biomarker result is significantly higher than that of a negative biomarker result, green shows that the hazard rate of a positive biomarker result is significantly lower than that of a negative biomarker result, gray shows that the difference between a positive and negative result is not significant. Immunohistochemistry biomarkers with the notable DNA excision repair protein ERCC1 are shown in the upper plot. Biomarkers derived from sequencing are displayed in the lower plot, and although there are only three sequencing markers, the epidermal growth factor receptor (EGFR) shows good prognostic ability.

The biomarkers that were used are compared between the matched and unmatched groups in Figure 1 (lower-left), and some were found to be prognostic for survival (Figure 3). Most were immunohistochemistry (IHC) based markers that have in previous cohorts been suggestive of response to cytotoxics, such as etoposide (TOPO1), platinum (ERCC1), and fluorouracil (TS). Sequencing data for *ALK* rearrangements and *EGFR* mutations were also included in these predictions. Only two patients in this dataset had PD-L1 testing using IHC: one patient tested positive, one negative.

## DISCUSSION

Here we looked at clinical outcome data for a lung cancer cohort provided by Caris Life Sciences, in their CODE database. Treatments predicted to be of benefit from tumor molecular profiling were proposed to clinicians, and the patients who received treatments that all agreed with them were compared to those whose therapies differed from the recommendations, i.e. they had at least one drug that was expected to lack benefit. The biomarkers profile was generally IHC-based in this cohort, and as such drew on evidence from previous studies that associated levels of key proteins in the tumor with responses to particular classes of cytotoxics, e.g. thymidylate synthetase levels with fluorouracil (5FU) response. Mutational analyses were restricted to a panel of 45 genes such as *EGFR*, *ALK*, *KRAS*, *NOTCH1*, *PIK3CA*, *PTEN* and *TP53*. Comparing the two groups indicated that the matched group had an increase of 5% in survival compared to an unmatched group on average, and an increase of 20 days i.e. 402 versus 382 days. There was therefore a trend towards increased survival in the matched group, but this was not statistically significant. It would be interesting to know if any patients had more than one Caris test performed sequentially after progression, and how the results compared, but this information was not available in the dataset that we used.

The unmatched group received 0.4 more therapies than the matched group on average, and survived for less time. This could be because the unmatched group had tumors that were more advanced than those from the matched group (e.g. 26 vs 17 stage IV tumors respectively-see Table 2). The unmatched group adhered less to the recommended treatments largely due to the use of platinums. This is likely because platinum-based doublets remain standard evidence-based therapeutics in NSCLC, with clinicians seeing them as a backbone of therapy. Practitioners are therefore reluctant to discard these agents, even in the presence of a molecular profile suggesting carboplatin may be less efficacious in a particular patient, e.g. given loss of *ERCC1* expression. This may reflect the controversy and mixed reports surrounding this biomarker, with recent data showing unclear prognostic significance [6].

As this panel was historical, many notable prognostic biomarkers that would be considered useful today, such as *FGFR1* or *FGFR3* amplification, *RET*, *MET*, *DDR*, *KRAS*, *BRAF* and *PIK3CA*, were not included for the selection of targeted therapy in the NSCLC cohort. All of these oncogenes have potential associated therapeutic treatments. Indeed a large proof of concept phase 3 study in France that used a panel including the majority of the above genes to stratify over seventeen thousand patients with NSCLC, showed that about half of all patients had a mutation in one of the above genes, and these patients had better response rates to first and second line treatments, and better survival [7].

Overall this study shows limited benefits from performing IHC-based profiling of NSCLC patients to determine response to cytotoxic chemotherapy. It is clear that clinicians rely heavily on cytotoxics when no molecularly targeted agent is appropriate, and are reluctant to implement the suggested regimens on the basis of IHC-based molecular markers that have had at best mixed evidence. We see a lack of difference in survival of non-small cell lung cancer patients according to treatment selection in this molecular characterization cohort. This may be due to the small sample size and limited number of validated biomarkers being explored in the biomarker panel here. In this cohort of patients, ERCC1 loss was predictive of poor survival (Figure 3), and we would recommend extending routine profiling in NSCLC patients to include clinically actionable driver oncogenes that have been demonstrated to enable selection of appropriate targeted therapies beyond cytotoxic chemotherapy.

## METHODS

The Caris CODE database (Comprehensive Oncology Database Explorer) contains tumor molecular profile data for 841 patients with solid tumors (CODE version 1.0). It also contains demographic information about these patients, the drug treatments that they received before and after molecular profiling and records of their clinical outcomes while they were still being monitored. There are 91 lung non-small cell lung cancer patients within this database, and this lung cancer cohort was mined after web scraping to extract the data from their website, to understand if molecular characterization affected drug selection by treating physicians, and if any molecular subsets had different outcomes across tumor types. Tables 1 and 2 describe the clinical characteristics of the patients that were profiled. According to Caris, 46% of patients had a metastatic sample profiled in this dataset.

Although the amount of time that patients were monitored varied-as shown in Figure 2-on average patients’ treatment records were available for 654 days after diagnosis (737 for matched treatment patients, 583 for unmatched). The time of monitoring after profiling was 391 days on average, and the longest period of monitoring after profiling was 1683 days (the patient represented on the furthest right of Figure 2) which was 1734 days after diagnosis. The longest amount of time that treatment records were available after diagnosis was 5242 days.

## Abbreviations

5FU: fluorouracil
IHC: immunohistochemistry
NSCLC: non-small cell lung cancer
OS: overall survival
